# Dislocation of the Hip into the Foramen Thyroideum—Reduction at the End of Six Weeks

**Published:** 1880-09

**Authors:** Herman Mynter


### DISLOCATION OF THE HIP INTO THE FORAMEN THYROIDEUM—REDUCTION AT THE END OF SIX WEEKS.

BY HERMAN MYNTER, M. D.

Lizzie Zehnder, a healthy and well-developed girl, 15 years of age, slipped with her left foot on going down-stairs, by which her leg was brought into a state of extreme abduction, while her body fell forward. She missed two steps of the stairs, but did not fall to the ground. She heard a noise in the left hip and experienced an acute pain, but was able to walk afterwards with the aid of crutches, although limping considerably. There was, at first, considerable swelling around the joint, but this disappeared. Fourteen days afterwards an irregular physician was called in, who pronounced the injury rheumatism. He made some movements of the joint, after which the pain increased and the walking became more impaired so that she was obliged to remain in bed.

July 22, 1880, six weeks after the accident, Dr. Tobie was called in, and diagnosticating a dislocation forwards into the thyroid foramen, (foramen obturatorium,) turned the patient over to me for surgical treatment. On examination the left lower extremity was seen to be elongated, adducted, slightly rotated outwards and flexed in the hip-joint. It could in some measure be abducted and rotated outwards, but not abducted and rotated inwards. Around the hip a marked deformity was seen, instead of the rounding of the hip a slight concavity being found. The trochanter major had seemingly disappeared, and was felt in a vertical line with the anterior superior spine of the ileum. By slight movements of the foot the head of the femur could be felt indistinctly in the thyroid foramen, and by exploration through the rectum something could be felt moving on the outer side of the membrana obturatoria, when the leg was rotated. The walk was characteristic, the left foot being held in front of the healthy one and could not be brought behind it. The measurement from anterior superior spine of the ileum to malleolus internus showed but about ¾ inches lengthening on the left side (although the knee seemed two inches lower) measurement from symphysis pubis, the right leg being abducted and rotated as much as the left, showed 1½ inches lengthening for the left side. The pelvis was tilted about one inch on the left side.

July 23d. Reduction was attempted under ether, Drs. Tobie, Hauenstein, Gay, Lothrop, Diehl, Barker and Peterson being present and assisting. The movements used were 1st, abduction, followed by flexion to a little more than a right angle, adduction to the vertical line, rotation inwards, the leg at the same time being brought down; and 2d, rotation outwards, adduction and the knee being brought down below the right knee. Pulleys were used and considerable power expended. Attempts were made for about 1½ hour, but at no time a characteristic slipping in was felt. After that time the form of the hip seemed normal. The trochanter major occupied its natural position and the rounding of the hip had returned. Measurement from spina ilei anterior superior to top of trochanter major was four inches on both sides, and when the patient was lying on her face, the measurement from trochanter to the middle line was seven inches on both sides. Measurement to malleolus internus was the same on both sides, but the pelvis was still tilted one inch, and the limb was apparently elongated one inch.

The head could no longer be felt in the thyroid foramen, neither outside nor in the rectum; the leg could be moved in all directions as much as the right (adduction excepted, where there was a slight difference.) The patient was put to bed and an ice-bladder applied over the hip. The farther progress was favorable. She staid in bed fourteen days, the limb being moved daily, and August 14th, treatment being discontinued, she walks as well as ever and can move her left limb in all directions as much as her right, but the pelvis is still tilted when she lies on her back, while it is not noticed when she walks.

This case is of interest on account of the severe injury following a slight accident and of the perfect recovery obtained after a lapse of six weeks. The continuation at the tilting of the pelvis after the recovery shows, that too much stress has been laid on this symptom. To be sure, the tilting may have been present before the injury, although it has never been noticed either by the parents or the patient.

